# Neural Decoding of Spontaneous Overt and Intended Speech

**DOI:** 10.1044/2024_JSLHR-24-00046

**Published:** 2024-08-06

**Authors:** Debadatta Dash, Paul Ferrari, Jun Wang

**Affiliations:** a Department of Neurology, The University of Texas at Austin; b Helen DeVos Children's Hospital, Corewell Health, Grand Rapids, MI; c Department of Speech, Language, and Hearing Sciences, The University of Texas at Austin

## Abstract

**Purpose:**

The aim of this study was to decode intended and overt speech from neuromagnetic signals while the participants performed spontaneous overt speech tasks without cues or prompts (stimuli).

**Method:**

Magnetoencephalography (MEG), a noninvasive neuroimaging technique, was used to collect neural signals from seven healthy adult English speakers performing spontaneous, overt speech tasks. The participants randomly spoke the words yes or no at a self-paced rate without cues. Two machine learning models, namely, linear discriminant analysis (LDA) and one-dimensional convolutional neural network (1D CNN), were employed to classify the two words from the recorded MEG signals.

**Results:**

LDA and 1D CNN achieved average decoding accuracies of 79.02% and 90.40%, respectively, in decoding overt speech, significantly surpassing the chance level (50%). The accuracy for decoding intended speech was 67.19% using 1D CNN.

**Conclusions:**

This study showcases the possibility of decoding spontaneous overt and intended speech directly from neural signals in the absence of perceptual interference. We believe that these findings make a steady step toward the future spontaneous speech-based brain–computer interface.

Locked-in syndrome, a condition resulting from brain damage or late-stage amyotrophic lateral sclerosis (ALS), leaves individuals completely paralyzed, yet cognitively aware. Traditional speech communication becomes impossible, due to the total loss of motor and speech abilities (Kohnen et al., 2013; Laureys et al., 2005; Smith & Delargy, 2005). Some patients retain facial muscle twitches, upper eyelid control, and vertical eye movement, which could facilitate nonverbal communication (Smith & Delargy, 2005). However, for patients in completely locked-in state, the brain is the only source of communication, where a brain–computer interface (BCI) can play a role. Currently, the primary solution for providing communication access to these patients lies in commercially available electroencephalography (EEG)–based BCI spellers (Rezeika et al., 2018). Unfortunately, these spellers are notably sluggish producing no more than 10 words per minute, failing to deliver a satisfactory communication experience (Kundu & Ari, 2022; Rezeika et al., 2018). Recent improvements using imagining handwriting of letters (Willett et al., 2021) or imagining letters directly further improved the communication rate to about 15 words per minute (Moses et al., 2021). Still, in contrast, the typical speech rate in our daily life is up to 200 words per minute. The promising avenue of neural speech decoding holds potential for a more effective solution. By directly mapping neural signals to speech, neural speech decoding has the capacity to restore a more natural and efficient means of communication (Anumanchipalli et al., 2019; Dash et al., 2020a; Iljina et al., 2017; Makin et al., 2021; Moses et al., 2019, 2021).

The most compelling investigations into the direct mapping of neural activity to speech or text have utilized invasive techniques like electrocorticography (ECoG; Derix et al., 2014; Herff & Schultz, 2016; Kanas, Mporas, Benz, Huang, et al., 2014; Kanas, Mporas, Benz, Sgarbas, et al., 2014; Ramsey et al., 2018; Song et al., 2014; Towle et al., 2008) or stereo-electroencephalography (sEEG; Herff, Diener, et al., 2020; Herff, Krusienski, & Kubben, 2020). Although these approaches show promise, they require neurosurgery to implant electrodes on the surface of or within the brain. Thus, their invasive nature may deter many patients, limiting their scalability. In contrast, noninvasive neuroimaging modalities such as functional magnetic resonance imaging (fMRI), functional near infrared spectroscopy (fNIRS), surface EEG, and magnetoencephalography (MEG) have emerged in the field of speech decoding. fMRI and fNIRS measure microvascular hemodynamic responses related to brain activity and have a temporal resolution in the order of seconds. EEG and MEG, on the other hand, measure electric and magnetic fields, respectively, associated with intracellular postsynaptic neuronal currents at a temporal resolution that matches the speed of brain activity. Although not yet attaining the precision of invasive methods, noninvasive neural speech decoding has demonstrated accurate classification of closed-set vocabularies, encompassing phrases (Dash et al., 2020a; Dash, Ferrari, Berstis, & Wang, 2021), words (D'Zmura et al., 2009; García-Salinas et al., 2019; Panachakel & Ramakrishnan, 2021; Porbadnigk et al., 2009), and even phonemes (Chi et al., 2011; Sakai et al., 2021; Suyuncheva et al., 2021). These encouraging findings suggest that noninvasive speech-BCI holds the potential, and perhaps preference, for restoring speech communication in individuals facing conditions like locked-in syndrome.

MEG has distinctive advantages compared to other noninvasive neuroimaging modalities for investigating neural speech decoding. Unlike EEG-recorded electrical signals, MEG-recorded magnetic field signals pass through the dura, skull, and scalp with minimal distortion. This characteristic enables MEG to offer a more precise depiction of underlying brain activities. MEG has a higher spatial resolution than EEG or fNIRS and superior temporal resolution (1 ms or even lower) compared to fMRI (Cohen & Cuffin, 1983). Recent MEG-based speech studies suggest the efficacy of MEG in capturing the fast temporal dynamics of the speech signal (Dash et al., 2020b; Dash, Ferrari, Berstis, & Wang, 2021; Memarian et al., 2012; Simos et al., 1998) and provide further evidence in support of the use of neuromagnetic signals in speech decoding. Moreover, advancements in MEG device technology, such as optically pumped magnetometers (Boto et al., 2017, 2018; de Lange et al., 2021; Livanainen et al., 2023; Roberts et al., 2019; Tierney et al., 2019), have paved the way for the development of next-generation, wearable MEG devices, addressing the limitations of nonportability and cost associated with traditional MEG machines. These unique benefits make MEG a great fit for the investigation of spontaneous speech decoding.

Currently, most neural speech decoding studies hinge on overt responses to cued stimuli, such as picture naming (Levelt et al., 1998) or overt reading (Anumanchipalli et al., 2019; Dash et al., 2020a). In the realm of noninvasive whole-scalp modalities, the distinction between the decoder identifying task effects related to the stimulus and decoding genuine speech-motor activation remains unclear. Commonly, yes or no stimuli are employed in speech decoding studies (Balaji et al., 2017; Chaudhary et al., 2017; Gallegos-Ayala et al., 2014; Hashim et al., 2018; Hwang et al., 2016; Rezazadeh Sereshkeh et al., 2017, 2019), yet the precise nature of what is being decoded—whether it's the speech activity itself or the brain state linked to the stimulus processing or task context—is not well defined. This marks a notable hurdle for the development of speech-BCIs for decoding spontaneous speech using noninvasive methods. Furthermore, for patients with difficulty in articulation or with articulatory paralysis, speech-BCI technology should focus on intended or imagined speech decoding rather than overt speech, for which the current technology is limited. Although a few studies (Brumberg et al., 2011; Dash, Ferrari, Berstis, & Wang, 2021; Dolcos & Albarracin, 2014; Hwang et al., 2016) have attempted to decode intended/imagined speech, the decoding performance has been relatively poor compared to overt speech decoding.

To address these challenges, we collected simultaneous MEG and speech recordings from participants, as they randomly spoke yes or no at a self-paced rate in the absence of any cue and attempted to decode speech information directly from the MEG signals. This design removes the contextual stimulus information common in typical experimental designs allowing for decoding of brain responses more related to spontaneous speech motor articulation. The choice of yes and no as stimuli was intentional, given their relevance in designing speech-BCIs for effective Q&A–based communication (Hill et al., 2014; Huang et al., 2021). Classification of the yes or no intended speech, overt speech, and postspeech processing was performed by training machine learning classifiers on time-locked neuromagnetic signals. Importantly, decoding during these three task stages allows us the ability to benchmark spontaneous speech decoding against the other stages that are commonly embedded in traditional task designs.

## Method

### Participants

Eight healthy adult participants (five females, 22–30 years of age) without any known neurological conditions participated in the experiment. The participants had normal or corrected to normal vision. No speech/language/hearing or cognitive history was reported from the participants. All participants were English speakers. All participants provided informed consent in compliance with the University of Texas at Austin institutional review board (IRB 2015-09-0042).

### Protocol

**Figure 1.**
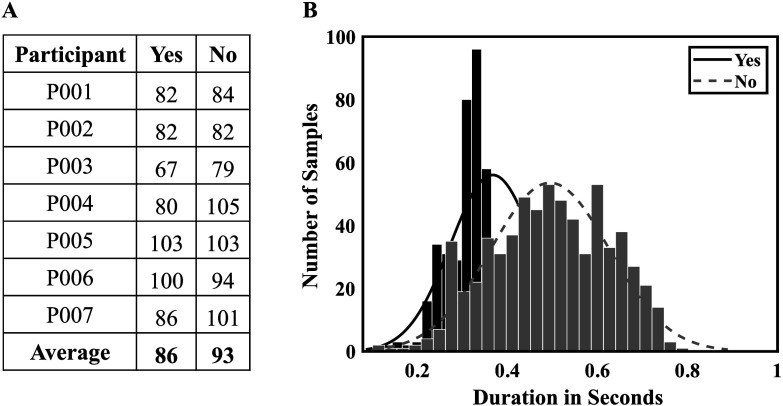
Data summary. (A) Number of spontaneous speech samples (yes or no) uttered by each participant. (B) The distribution of yes or no speech duration across all participants is illustrated in the figure below. Notably, there was no statistically significant difference observed between the durations of yes and no overt speech.

Participants were comfortably seated within the MEG unit with their arms resting on a table and overtly pronounced the words yes and no randomly at a rate of approximately one word per 4–5 s. Participants were explicitly instructed not to alternate back and forth between the two words but rather randomly sample between them. An example of a sequence would be yes, yes, no, no, no, yes, no, no, yes … no. An experimenter first demonstrated the task, and the participants practiced the randomization prior to starting the experiment. During the experiment, the researcher kept a running tally of each exemplar. The task condition ended when a minimum of 80 repetitions for each word was achieved (see Figure 1A). We also checked the trial length and observed that yes and no trials are of no significant difference in duration (equivalence testing [Lakens et al., 2018]: *p* < .05, *df* = 1198, effect size = 0.16), which eliminated the potential duration effects in this study (see Figure 1B).

### Data Collection

Participants' neuromagnetic brain activity was recorded during the task using a 306-channel (204 gradiometers and 102 magnetometers) MEGIN TRIUX (MEGIN LLC US) inside a two-layer magnetically shielded room. The MEG signals were recorded at a 4 kHz sampling rate with an online hardware filter of 0.03–1333 Hz. Two pairs of bipolar surface electrodes were used to record the electrocardiogram and the electrooculogram (EOG) signals. Voice data were recorded into the MEG system's analog to digital channels using a lapel microphone with the battery and transducer sitting outside the shielded room to avoid significant artifacts.

### Data Preprocessing

First, the MEG data were visually inspected for significant artifacts. Eye blinks and muscle artifacts were marked. The data were then low-pass filtered below 250 Hz with a fourth order noncausal Butterworth filter and notch filtered with cutoff frequencies at 60 Hz and its harmonics. One sensor that displayed a flat or noisy response was omitted from the subsequent analysis. Independent component analysis (ICA) was employed to eliminate artifacts arising from eye blinks, eye movements, and heartbeats. The effectiveness of the ICA procedure was validated by inspection of the data at the artifact markings, EOG, and ECG. During this processing, data from one participant contained continuous artifacts and were removed from the analysis leaving seven participants for analysis.

Second, to facilitate MEG data epoching, the speech signal was extracted and denoised using a Wiener filter (Plapous et al., 2006). Voice activity detection (VAD; Sohn et al., 1999) was implemented to pinpoint the acoustic onset and offset of the words. The duration of each speech event was attached to the VAD labels. These labels were manually inspected, and corrections were made to ensure accurate marking. Each trial was categorized into either the yes or no class after listening to the acoustic signals. Finally, the MEG data were epoched into trials around the speech signal. Each trial was then manually segmented into three segments that are of equivalent length, pre-speech (intended speech), (overt) speech, and postspeech. Here, we define overt speech period as the time segment during the speech signal, that is, from acoustic onset to acoustic offset, intended speech period as the time segment with the same length as that of overt speech segment before the acoustic onset and postspeech period as the time segment with the same length with that of overt speech segment after the acoustic offset. There was no overlap between postspeech of a previous trial and pre-speech of a current trial. The onset and offset of overt speech segments were evaluated based on synchronously collected acoustic data. Only the MEG gradiometers were used for decoding analysis, as our prior studies indicated that gradiometers have more robust information and outperform magnetometers (Dash, Ferrari, Babajani-Feremi, et al., 2021)

### Experimental Setup and Decoding Methods

**Figure 2.**
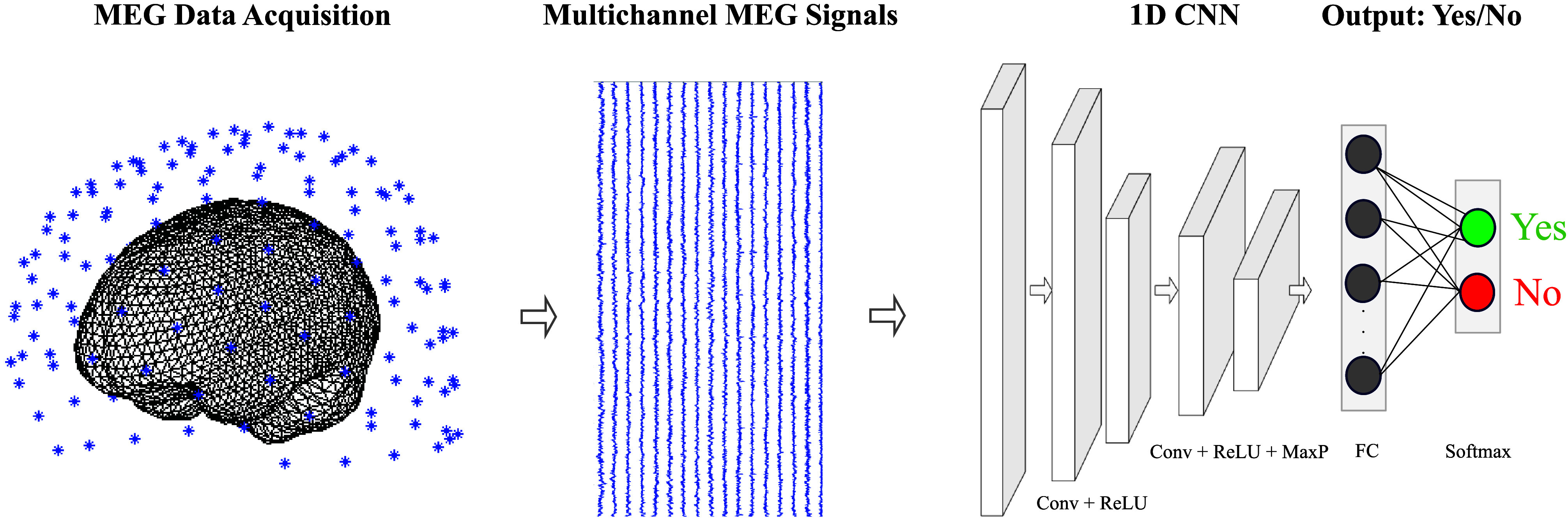
Pipeline for spontaneous speech decoding. Magnetoencephalography signals were epoched to yes or no segments corresponding to the yes or no speech. After a baseline linear discriminant analysis decoder training for yes or no classification, a one-dimensional (1D) convolutional neural network model was trained that consisted of two blocks of 1D convolution layers and Rectified Linear Unit (ReLU) layers, followed by global average max pooling, fully connected layers, and finally a softmax layer that resulted the probability of a given sample belonging to class yes or no. MEG = magnetoencephalography; CNN = convolutional neural network; Conv = convolution; MaxP = Max Pooling; FC = Fully Connected.

To optimize model training and evaluation, we split the data allocating the first 70% of trials for training and the last 30% of the trials for testing to preserve the temporal order of the MEG time series data and transferability to online decoding. The training was performed for each participant using a fourfold cross-validation (CV) strategy to find the optimal model configuration with tuned hyperparameters. The choice of fourfold CV was to ensure a minimum of 40 trials (80 total trials per class × 70% for training × 75% training data for fourfold CV, that is, threefold for training and onefold for validation in each of the four iterations of fourfold CV) in the training fold based on our previous research in determining optimal number of trials for decoding (Dash, Ferrari, Malik, Montillo, et al., 2018). To enhance robustness, the fourfold CV was repeated 10 times through Bayesian bootstrapping (Rubin, 1981). The performance metric was decoding accuracy, calculated as the ratio of correct predictions by the decoders to the total number of predictions. The CV performance was computed by averaging the accuracy across the fourfold and the 10 bootstrap runs. The trained decoder's predictions on the test data were then reported as the final decoding performance. We performed yes and no classification using linear discriminant analysis (LDA) and one-dimensional convolutional neural networks (1D CNN) for both intended and overt speech. Figure 2 illustrates the classification pipeline.

#### LDA for “Yes” or “No” Classification

Initially, we trained an LDA model as our baseline decoder. LDA, a supervised machine learning classifier, identifies directions (“linear discriminants”) that maximize the separation between multiple classes (McLachlan, 2005). In our past neural speech decoding studies, LDA demonstrated performance equivalent to support vector machines and multilayer perceptron classifiers (Dash, Ferrari, Babajani-Feremi, et al., 2021; Dash, Ferrari, & Wang, 2021) and outperformed naïve Bayes, decision trees, ensembles, and k-nearest neighbor classifiers (Dash et al., 2020). Due to its relatively fast training and minimal hyperparameter tuning, LDA became our preferred baseline decoder. We fine-tuned the classifier's hyperparameters (alpha and beta parameters of the Dirichlet distribution) for each participant using Bayesian optimization that works by considering the previously seen hyperparameter combinations when determining the next set of hyperparameters to evaluate (Wu et al., 2019). Root mean square features extracted from MEG signals were employed to train the LDA decoder, given their proven effectiveness in MEG- and EEG-based decoding analyses (Dash, Ferrari, Babajani-Feremi, et al., 2021; Sereshkeh et al., 2017; L. Wang et al., 2020). The feature dimension was 203 corresponding to the 203 gradiometer sensors.

#### 1D CNN for Yes or No Classification

1D CNNs are particularly well suited for tasks involving sequential data, such as time series analysis, speech recognition, and natural language processing (Kiranyaz et al., 2019). 1D CNNs consist of convolutional layers (filters or kernels to local regions of the input sequence, capturing patterns and features to learn hierarchical representations of the input data), pooling layers (downsample the spatial dimensions of the input, reducing the computational complexity of the model and aiding in the extraction of important features), a flatten layer (convert the high-dimensional output into a 1D vector), which can then be fed into fully connected layers for further processing. Convolutional operations provide a degree of translation invariance, allowing the model to recognize patterns regardless of their position in the input sequence. The use of shared weights in convolutional layers enables the model to learn spatial hierarchies efficiently.

We used a 1D CNN that consisted of two blocks of 1D convolution layer, a ReLU layer, and a normalization layer. The 1D convolution layers had 32 and 64 filters of size 5. They were followed by a global average pooling layer and then a flatten layer and a fully connected layer of two nodes. The last layer was a softmax layer with two nodes depicting the output probability of the two classes (yes and no). An Adaptive Moment Estimation optimizer was used to train the model with initial learning rate (0.003–0.006: different for each participant) with 100 maximum number of epochs. A grid search strategy was used to tune the hyperparameters of the model.

## Results

**Figure 3.**
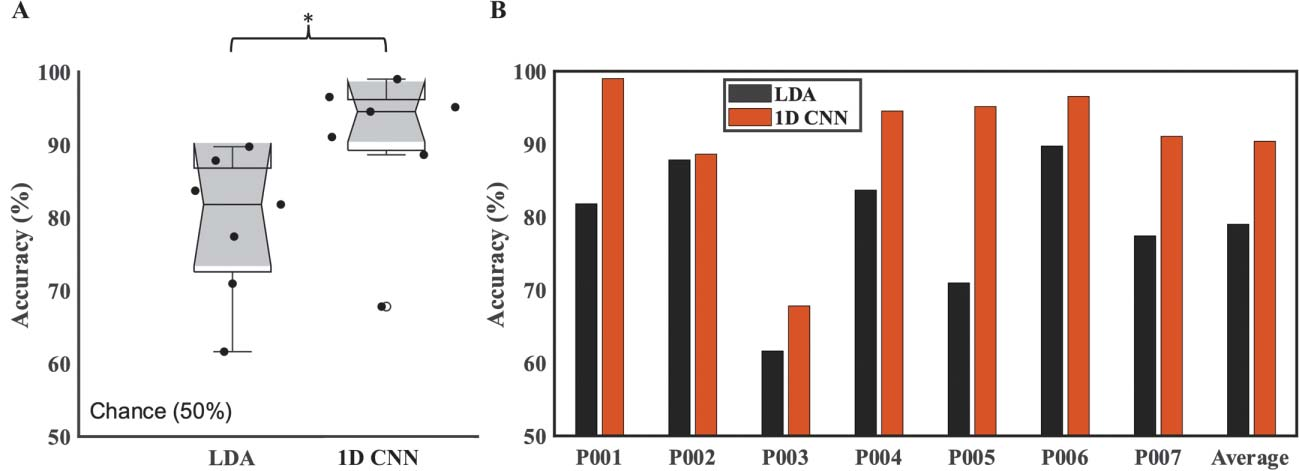
Spontaneous overt speech decoding. (A) Comparison of spontaneous overt speech decoding performance between baseline LDA decoder and one-dimensional convolutional neural network (1D CNN). Note that 1D CNN decoder achieved about 90% decoding accuracy, significantly higher than chance level and baseline decoder performance. (B) Decoding performances for individual participants are shown. Note that even the participant with least decoding accuracy also showed higher than chance level performance. LDA = linear discriminant analysis. *p < .001.

The decoding of spontaneous overt speech yielded an accuracy of 79.02% ± 3.75% with LDA and 90.40% ± 3.98% with 1D CNN (see Figure 3A). Both LDA and 1D CNN decoding performances surpassed the chance level of 50% (LDA: *t* = 21.05, *p* < .001; 1D CNN: *t* = 22.72, *p* < .001). Even the participant with the lowest performance (P003: LDA: 61.64%; 1D CNN: 67.82%) achieved results significantly higher than chance level. Notably, this participant also had the fewest samples among all participants (refer to Figure 1A). Furthermore, the average performance with 1D CNN was significantly higher than that with baseline LDA (*t* = 3.87; *p* = .008). These results also suggested CNN outperformed LDA in neural speech decoding. We also found CNN outperformed other machine learning classifiers such as artificial neural network in our prior studies (Dash et al., 2020a). Thus, we applied CNN only for intended speech decoding.

**Figure 4.**
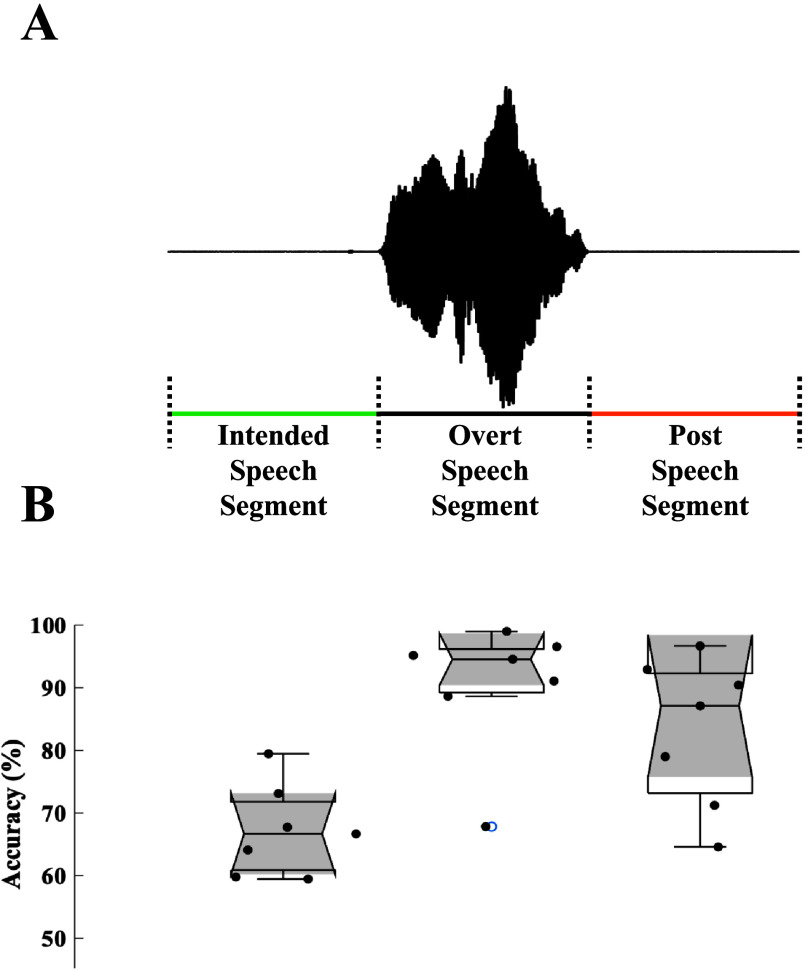
Spontaneous intented speech decoding. (A) Magnetoencephalography data prior to the acoustic onset (intended speech segment), from acoustic onset to acoustic offset (overt speech segment), and after acoustic offset (postspeech segment) were used to decode spontaneous speech. (B) Decoding performance with one-dimensional convolutional neural network resulted in about 67% decoding accuracy for intended speech significantly above chance level. Decoding accuracies for overt speech and postspeech was higher than intended speech.

The mean decoding accuracy for intended speech was 67.19% ± 2.72% using 1D CNN, significantly above chance level (*t* = 24.72, *p* < .001; Figure 4A). Intended speech decoding performance was lower compared to decoding overt speech as well as decoding postspeech (i.e., with taking data after acoustic offset; one-way ANOVA: *F* = 9.72, *p* = .0014; post hoc Tukey tests: intended vs. overt- *p* = .0012; intended vs. post- *p* = .0217).

## Discussion

Our study demonstrated the feasibility of decoding spontaneous speech with high accuracy using noninvasive neuromagnetic signals. Importantly, our results revealed that not only spontaneous overt speech but also spontaneous intended speech can be decoded from neural signals.

Utilizing a spontaneous speech paradigm removed potential contamination by cue-based interference such as task effects or perceptual processing and provided solid support toward being able to interpret genuine speech motor activity from the brain. Much of the existing literature on overt speech decoding involves tasks with auditory or visual stimuli that participants are required to recognize, process, and respond to such as picture naming (X.-J. Wang, 2010) or paragraph reading (Anumanchipalli et al., 2019). In such scenarios, it becomes challenging to discern whether the neural activity being decoded solely corresponds to overt speech processes or incorporates information related to stimulus recognition and processing, leading to ambiguity. Although some studies have addressed this concern by implementing delayed overt speech production protocols introducing a 1–3 s delay between stimulus presentation and speech production (Dash et al., 2020a), the lingering effects of postauditory or visual processing in the brain may persist into the overt speech production stage. In our study, participants spontaneously uttered yes or no without any explicit cue thereby avoiding these effects.

It is important to acknowledge that overt speech decoding can potentially be influenced by movement artifacts. This poses a twofold problem for decoding. First, high-frequency muscle artifacts corrupt the MEG sensor data potentially masking neural signals and, secondly, the low-frequency noise components related to jaw and tongue movements potentially create spurious decoding. Importantly, in a previous study that incorporated movement tracking, we showed that overt speech decoding from MEG signals is clearly a combination of both neural and movement signals (Dash, Ferrari, Malik, & Wang, 2018). Despite the challenges, our study achieved high performance in classifying overt “yes” and “no” speech samples. This success strongly suggests that spontaneous overt speech can indeed be effectively decoded using non-invasive neural signals, showcasing the robustness of our approach.

When considering patients with locked-in syndrome, the focus shifts to decoding intended or imagined speech for speech-BCI applications. In studies utilizing fNIRS (Chaudhary et al., 2017; Gallegos-Ayala et al., 2014; Hwang et al., 2016; Rezazadeh Sereshkeh et al., 2019), yes or no intended or imagined speech decoding achieved 64%–75% performance. Similarly, with EEG (Balaji et al., 2017; Hashim et al., 2018; Rezazadeh Sereshkeh et al., 2017), yes or no classification resulted in 63%–73% decoding performance. Evidently, decoding intended or imagined speech poses challenges compared to overt speech decoding. In our study, we achieved 67.19% accuracy in decoding intended speech, on par with current literature. Notably, our intended speech was spontaneous, adding an extra layer of difficulty in contrast to the previous literature that used visual or auditory stimuli for task presentation where cue-based task effects might contribute to decoding performance. This underscores the significance of our results in addressing the challenges associated with decoding spontaneous intended speech. We note that, although our spontaneous intended speech decoding avoids the contribution from cue-based task effects, motor artifacts present during our intended speech segment could have contributed to decoding performance. Furthermore, the intended speech decoding performance needs to be as accurate as the overt speech decoding performance, at the least, for an ideal speech-BCI use case in clinical populations, which necessitates further research with larger data set (e.g., more trials), improved data quality (e.g., going to source space from sensor space), and better decoder models (e.g., deep learning). Furthermore, it is necessary to validate these findings with data from clinical population (e.g., ALS) as those with neurological disease may have aberrant neural signals (Wilson & Schneck, 2020), resulting reduced decoding performance (Dash et al., 2020).

This capability of decoding intended speech holds great promise for applications involving decoding intended speech, especially in cases such as ALS or for locked-in patients. As anticipated, the most optimal performance was achieved during the overt speech production stage, where the decoder was trained exclusively with data captured between acoustic onset and offset. This stage encapsulates rich information, including speech motor articulation details, auditory feedback, and potential remainder movement artifacts. Although spontaneous intended speech decoding presents additional challenges, its necessity becomes apparent in the trajectory toward real-world speech-BCI applications.

Last and interestingly, we also obtained an above-chancel level accuracy for postspeech segment, which provides additional evidence that speech, like other task-based information processes, is not transient but dynamically linked to ongoing neural processes (for up to a few seconds). This further validates the drawback/limitation of using cue-based task designs to develop speech BCIs.

In sum, we demonstrated the feasibility of decoding spontaneous speech from noninvasive neural activity. Although challenging, spontaneous speech decoding is a step forward in decoding true speech motor activity from the brain, crucial for scalable speech-BCIs.

### Limitations

Our study utilized a small vocabulary set (yes and no) for spontaneous speech decoding. Starting with this limited set was crucial to demonstrate, for the first time, the feasibility of decoding spontaneous speech. Building on the success of this study, future studies are underway for spontaneous speech decoding with a larger close-set or even open-set vocabulary. Another limitation of our study is the choice of intended speech segment spanning up to acoustic onset. A previous EEG study (Ouyang et al., 2016) noted the presence of articulation artifacts at least 300 ms before voice/acoustic onset. Consequently, it is possible that these motor-related artifacts preceding the acoustic onset might have influenced the decoding performance in our study. A potential solution is to use kinematic onset (e.g., jaw movement) as overt speech onset, which will be investigated in future studies with an available data set.

## Data Availability Statement

The data are currently under processing for sharing in the future. Codes can be obtained from the corresponding author upon request.

## Notes

**Publisher Note:** This article is part of the Forum: Research Symposium on Artificial Intelligence.
